# Optimizing the Analytical Value of Oncology-Related Data Based on an In-Memory Analysis Layer: Development and Assessment of the Munich Online Comprehensive Cancer Analysis Platform

**DOI:** 10.2196/16533

**Published:** 2020-04-17

**Authors:** Daniel Nasseh, Sophie Schneiderbauer, Michael Lange, Diana Schweizer, Volker Heinemann, Claus Belka, Ranko Cadenovic, Laurence Buysse, Nicole Erickson, Michael Mueller, Karsten Kortuem, Maximilian Niyazi, Sebastian Marschner, Theres Fey

**Affiliations:** 1 Comprehensive Cancer Center Munich Munich Germany; 2 Comprehensive Cancer Center Ludwig-Maximilians-Universität München Munich Germany; 3 Comprehensive Cancer Center Technical University Munich Munich Germany; 4 German Cancer Consortium (DKTK, partner site Munich), German Cancer Research Center (DKFZ) Heidelberg Germany; 5 Department of Radiation Oncology University Hospital, LMU Munich Munich Germany; 6 Institute for Medical Information Processing, Biometry and Epidemiology Ludwig-Maximilians-Universität München Munich Germany; 7 University Eye Hospital Munich Munich Germany

**Keywords:** oncology, database management systems, data visualization, usability

## Abstract

**Background:**

Many comprehensive cancer centers incorporate tumor documentation software supplying structured information from the associated centers’ oncology patients for internal and external audit purposes. However, much of the documentation data included in these systems often remain unused and unknown by most of the clinicians at the sites.

**Objective:**

To improve access to such data for analytical purposes, a prerollout of an analysis layer based on the business intelligence software QlikView was implemented. This software allows for the real-time analysis and inspection of oncology-related data. The system is meant to increase access to the data while simultaneously providing tools for user-friendly real-time analytics.

**Methods:**

The system combines in-memory capabilities (based on QlikView software) with innovative techniques that compress the complexity of the data, consequently improving its readability as well as its accessibility for designated end users. Aside from the technical and conceptual components, the software’s implementation necessitated a complex system of permission and governance.

**Results:**

A continuously running system including daily updates with a user-friendly Web interface and real-time usage was established. This paper introduces its main components and major design ideas. A commented video summarizing and presenting the work can be found within the Multimedia Appendix.

**Conclusions:**

The system has been well-received by a focus group of physicians within an initial prerollout. Aside from improving data transparency, the system’s main benefits are its quality and process control capabilities, knowledge discovery, and hypothesis generation. Limitations such as run time, governance, or misinterpretation of data are considered.

## Introduction

In recent years, hospitals have been gradually transitioning from paper-based toward electronic documentation systems. The ongoing digitalization of routine data often results in the creation of large and comprehensive datasets, which can, under the right circumstances, open doors to further analysis and research [1,2]. These datasets are often considered to be “big data”, not always due to their size but also due to their complexity. Therefore, analysis of such large datasets, especially in cases of secondary use, is most often the biggest challenge [3-6]. Hence, a solution for managing and extracting the knowledge hidden in the raw data in a meaningful way for all potential end users is necessary [7]. Importantly, without proper tools and methods, interest in the data can decline and a dataset’s potential information content can remain unused and obscure. In general, standard statistical tools can be applied to tackle and analyze previously defined problems. However, smart business intelligence platforms such as Microsoft BI [8], QlikView [9], or, in a broader sense, SAP Hana [10] can allow for spontaneous and quick analysis of the data, and can therefore accelerate response time as well as facilitate response efforts. In 2012, a novel technology was introduced that utilizes such systems, termed “in-memory database.” This technology allows for the swift handling of data without investing too much effort in data preparation in contrast to online analytical processing systems [11-13]. Aside from alleviating daily tasks, allowing users to immediately dive into data further offers transparency, and can thus support knowledge discovery, hypothesis generation, and translational research [14,15]. Therefore, we decided to create a system utilizing an in-memory database based on the software QlikView.

The development was tested within a focus group of 10 physicians at the University Hospital of the Ludwig-Maximilians-University (LMU) in Munich. The system was set up together with its partnering site, the University Hospital of the Technical University Munich, Rechts der Isar, with both sites sharing the Cancer Retrieval Evaluation and Documentation System (CREDOS) as their local tumor documentation system. These systems allow the institutes to compile and track most oncology-relevant data [16], including specific information about diagnoses (eg, grading or histology), therapies, and follow up for most oncology patients that were treated in the centers. In total, the database contains more than 1000 attributes about the patients themselves, their medical history, and tumor descriptives. The software is suitable for not only a specific tumor site but also for all solid and nonsolid tumors, resulting in a large, complex, and comprehensive oncology database. The software was established at the local sites in 2010 and now includes most tumor entities. By the end of 2018, the databases contained detailed information for more than 20,000 patients at each site.

The primary purpose of the database is to measure specific key performance indicators such as summarizing the number of cancer cases of a specific organ, which thus serve as indicators of the eligibility of the sites to become, or remain, a certified center according to the German ONKOZERT guidelines [17]. Gathering and maintaining all of the necessary information is not a trivial task and requires resources. Therefore, collecting data solely for the purpose of certifying the centers might not be worth the effort. Consequently, to increase the usability of these big datasets so that they may be harvested for further purposes, we created an analysis layer that provides visual access to this complex dataset and offers easy-to-use tools, which allow end users to immerse into, and analyze, the data.

The analysis layer was recently (October 2018) rolled out and tested within the previously mentioned focus group at the Comprehensive Cancer Center Munich

(CCCM)-LMU site. We refer to this analysis platform as the Munich Online Comprehensive Cancer Analysis platform (MOCCA). This paper describes how the system was rolled out at our site and explains the major components of this analysis platform, and should thus serve as an inspiration for other institutions interested in making their data more accessible and transparent. Focusing on how to manage and organize large sets of oncology-related data, we present a variety of innovative ideas in terms of browsing, handling, and visualizing large cohorts of medical data, while addressing challenges that arose during the development.

## Methods

### Administrative Arrangements

To provide general access to the data, the first step was to set up a server within the clinical intranet, which continuously runs an instance of the QlikView Enterprise Edition. The administrative user interface of the Enterprise Edition allows for data loading routines. Thus, a daily routine was established and implemented, which imports the whole CREDOS dataset into the MOCCA system. QlikView was chosen because it comes with a toolbox, enabling the construction of the contents that can be saved within a proprietary data container (*.qvw file) [9]. The toolbox also offers an intuitive user interface and its own scripting (partly Structured Query Language-based) and data-handling language. The program files themselves can not only be viewed within the developer toolbox but can also automatically be transformed into a fully functioning website and Web view [18]. Thus, the developer does not have to create an HTML framework or any other Web content, but can rather concentrate on the contents’ objects themselves (eg, tables, graphs). End users can then comfortably access the resulting Web view via the URL and a Web browser without installing any additional software.

To control the Web access, we set up a connection to the hospital’s active directory using a lightweight directory access protocol (LDAP) [19]. Consequently, users are required to register with their hospital intranet login to view the contents but do not have to create new accounts or passwords. To conform with laws of privacy protection as well as issues of governance, users can only view contents that they are allowed to see according to the hospital’s policy. This assures that not every user who has access to the intranet can arbitrarily access the MOCCA platform. Instead, a user has to possess a single user license, which is a technical requirement before entering the platform. Detailed information about the permission system is described in the Permission System section below.

### Embedding the Tumor Documentation Data

**Figure 1:**
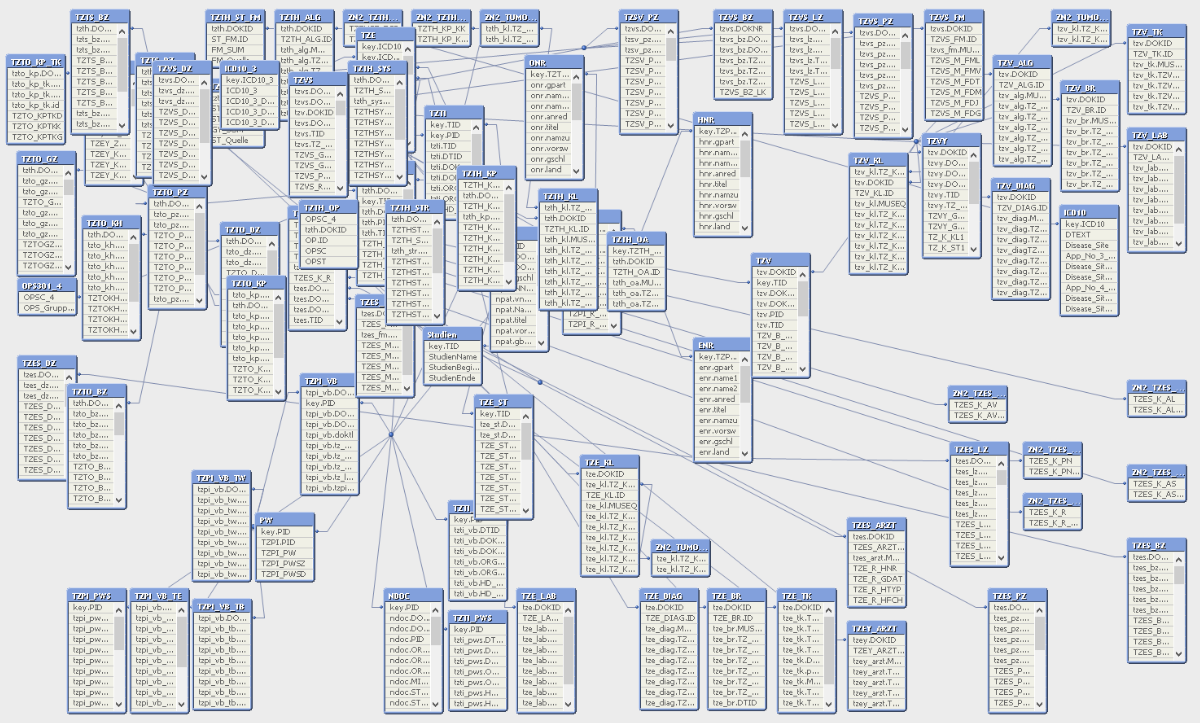
Screenshot of the current data model illustrating its complexity. The tables are framed in blue, displaying all of their included fields. The tables are linked via specifically chosen key attributes (eg, patient, tumor, or document ID).

The next major step involved in setting up the platform was the conversion of the CREDOS data model into a new QlikView data model. This involved interconnecting all tables by common, unique identifiers, similar to a standard relational database. We tried to stick as closely to the original data model of CREDOS as possible, and only incorporated changes when needed. When completed, this step enabled the system to fully unveil the in-memory database capabilities after loading the data. At this point, an end user can select, via a mouse click, a specific data field within the Web view (eg, male or female within the gender field). After selecting the relevant data field, all other data tables react in real time and restrict the contents to the selected cohort. In this way, it is possible, in a matter of seconds, to create different data cohorts constricted by arbitrary filters. Most normal business intelligence or query systems offer query interfaces where different filters can be applied. However, this interface is not necessary in QlikView since all data tables and fields displayed within the Web view can directly be used as a cohort filter. QlikView offers an additional functionality through which it shows the data model. A current view of the data model in our latest release is shown in Figure 1, which highlights the complexity of what is involved in this seemingly “simple” step.

We first organized both the data and the analysis layer in the same *.qvw data file. Subsequently, we decided to strictly separate the data modeling and analysis contents into two segregated QV files. The primary cleaning and preprocessing of the CREDOS data model into QlikView is done in the base module (base.qvw), while more advanced preprocessing as well as further analyses are done in the second module (analysis.qvw). This allowed us to simultaneously improve the data model along with the analytical tools and modules.

### Creating the Analytics Frontend

As mentioned above, the CREDOS data model is complex, comprising more than 1000 individual data fields, which themselves can have hundreds of different data values (eg, the data field diagnosis can have hundreds of different International Statistical Classification of Diseases and Related Health Problems [ICD]-10 codes [20]). As our main aim was to make this platform accessible for clinicians, innovative ideas on how to manage this large degree of complexity in an accessible and useful manner were necessary. We therefore structured the analysis tool into six data-related categories with their respective subcategories, which are listed in Textbox 1.

**Data-related categories and structure of the analysis tool.**

- First assessment
  - Diagnosis
  - TNM (tumor-node-metastasis; important tumor staging system)
  - Classifications

- Patient-based/related data
  - Cohort view
  - Single patient view

- Therapies
  - General information
  - Operations
  - Systemic therapies
  - Radiation

- Progression (follow up)
- Trial metadata
- Survival

For most categories (including subcategories), we implemented detailed and comprehensive views, including embedded tools that enable easily browsing and visualizing category-specific contents. The views were designed as different tabs, analogous to a standard website navigation bar (see Multimedia Appendix 1). Although extensive contents were created for most of these categories and subsequently included in the first version of the platform, the single patient view, as well as the trial metadata view, remain in a conceptual stage and have therefore been deactivated for the first release.

For each of the remaining categories shown in Textbox 1, our goal was to fill all related data attributes into a QlikView tab, which takes up one screen (optimized for a 1680 × 1050-pixel screen resolution). Owing to the overwhelming amount of data attributes, loading the tabs with data tables would have exhausted the pixel space within the tabs multiple times. Thus, to overcome the complexity of storing a lot of information within a small space while simultaneously ensuring that it appears in an easily comprehensible manner, we enabled the end user to hide or display specific data objects such as tables or graphs by utilizing variables, which are themselves controllable (eg, by buttons) within the front end.

**Figure 2:**
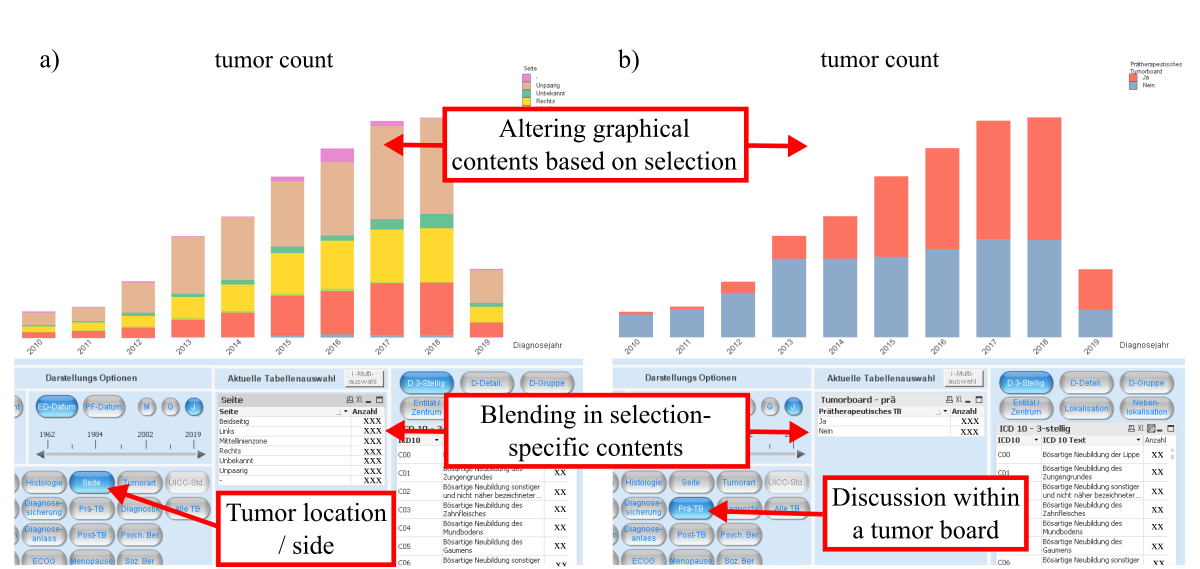
The image (screen language in German) displays two different selections within the first assessment (diagnosis) view. By simply clicking on different buttons, in this case “tumor location/side” in (a) and “discussion of a chosen case within a tumor board” in (b), it is possible to specifically select and examine the number of cancer cases with the chosen feature (in the chart stratified by date of diagnosis).

As shown in Figure 2, 18 buttons are displayed, which refer to 18 different data attributes related to the first diagnosis of a patient (eg, “vital status,” “histology,” or “side of tumor”). By clicking these buttons, a table showing the selected contents of these data fields as well as a corresponding graph are displayed. Although it would be possible to show the tables of all 18 attributes at the same time, we believe it makes more sense to provide control to the end users as to what they want to focus on. This results in a dramatic reduction in complexity of onscreen contents since most of the data are hidden at most times, and because users have an easier time reading the data they feel is relevant. This technique has been applied not only for this example but also for numerous contents within the entire platform. Figure 2 also illustrates how we utilized extensive numbers of easily readable charts to increase the end users’ experience by data visualization. As most data are interconnected within the data model, the integrated in-memory database allows the user to click in any chart directly, and instantly alters all other graphical objects and tables within the platform accordingly.

With regard to the graphical objects, in addition to reactive standard descriptive techniques such as bar charts or pie charts, we used a QlikView add-on (svgReader) [21], which allowed us to incorporate vector graphics and to color code them according to the selected data cohorts as well as underlying formulas such as relative abundance. Therefore, we were able to create innovative and interactive modules that increased the usability, readability, and interest of the data contents. For example, we created scalable vector graphics (SVGs) for the geographic locations of patients, a three-layer organ map including all oncology-relevant ICD-10 diagnosis codes, a map displaying the spread of metastases, and a two-layer map showcasing the areas of radiation. Within the three-layer organ map, we specifically combined the SVG map technique with the content-hiding technique described above.

**Figure 3:**
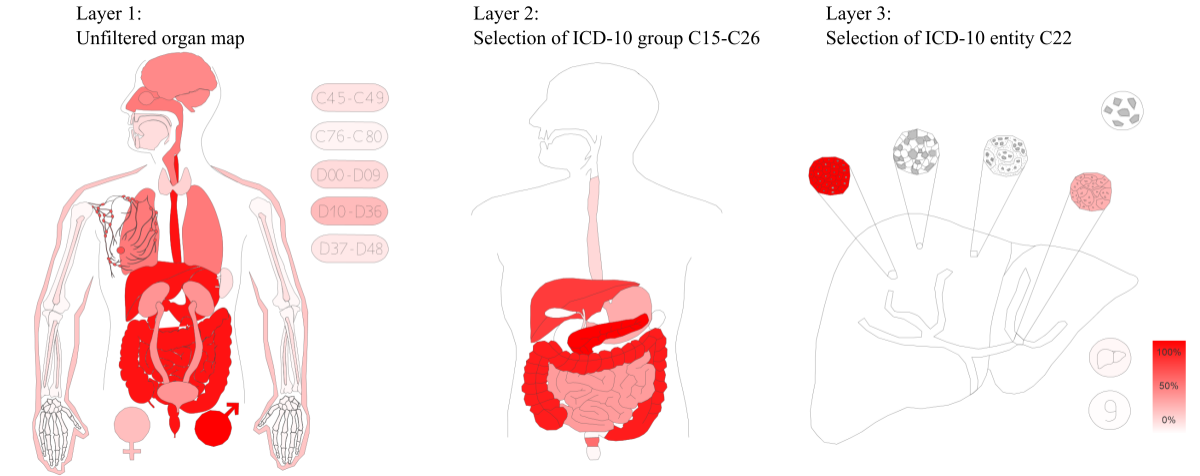
SVG-based organ map. Based on the diagnosis code (ICD-10), the map displays the relative amount of documented cases to each other as well as selected (via mouse clicks) ICD-10 groups (layer 2), or even specific organs (layer 3). In this example, the gastrointestinal subgroup had been selected by clicking within layer 1 (C15-C26), followed by clicking on the liver within layer 2, thereby restricting the cohort within module to C22* (liver) and displaying the relative abundance of affected segments within the liver (layer 3). A fully saturated color indicates the most commonly documented segments within the SVG, whereas lower levels of saturation linearly correspond to the amount of documentation for the associated segments. SVG: scalable vector graphic; ICD-10: International Statistical Classification of Diseases and Related Health Problems-10.

Figure 3 shows the first level of this three-level organ map. When a user clicks on a component (ie, an organ group) they switch to the specific ICD-10 group, which also sets a selection filter to this group for the whole platform. Accordingly, the image changes from the first layer to the chosen ICD-10 group. From this second layer of detail, the user can select a single organ (third layer), which then displays the components of the organ. The segments of the graphics are colored according to the relative abundance of tumor cases; for example, Figure 3 layer 1 shows that gastrointestinal tumors were predominantly treated (or rather documented) at our hospital. In general, for all of these maps, a more saturated color indicates a higher degree of documentation (linear in proportion to the data). In other words, if an organ consisted of two segments and only one segment had 100 documented cases while the other segment only had 50 documented cases within the system, the first segment would be fully color-saturated (100% on the red scale), while the other segment would only be color-saturated by 50%.

**Figure 4:**
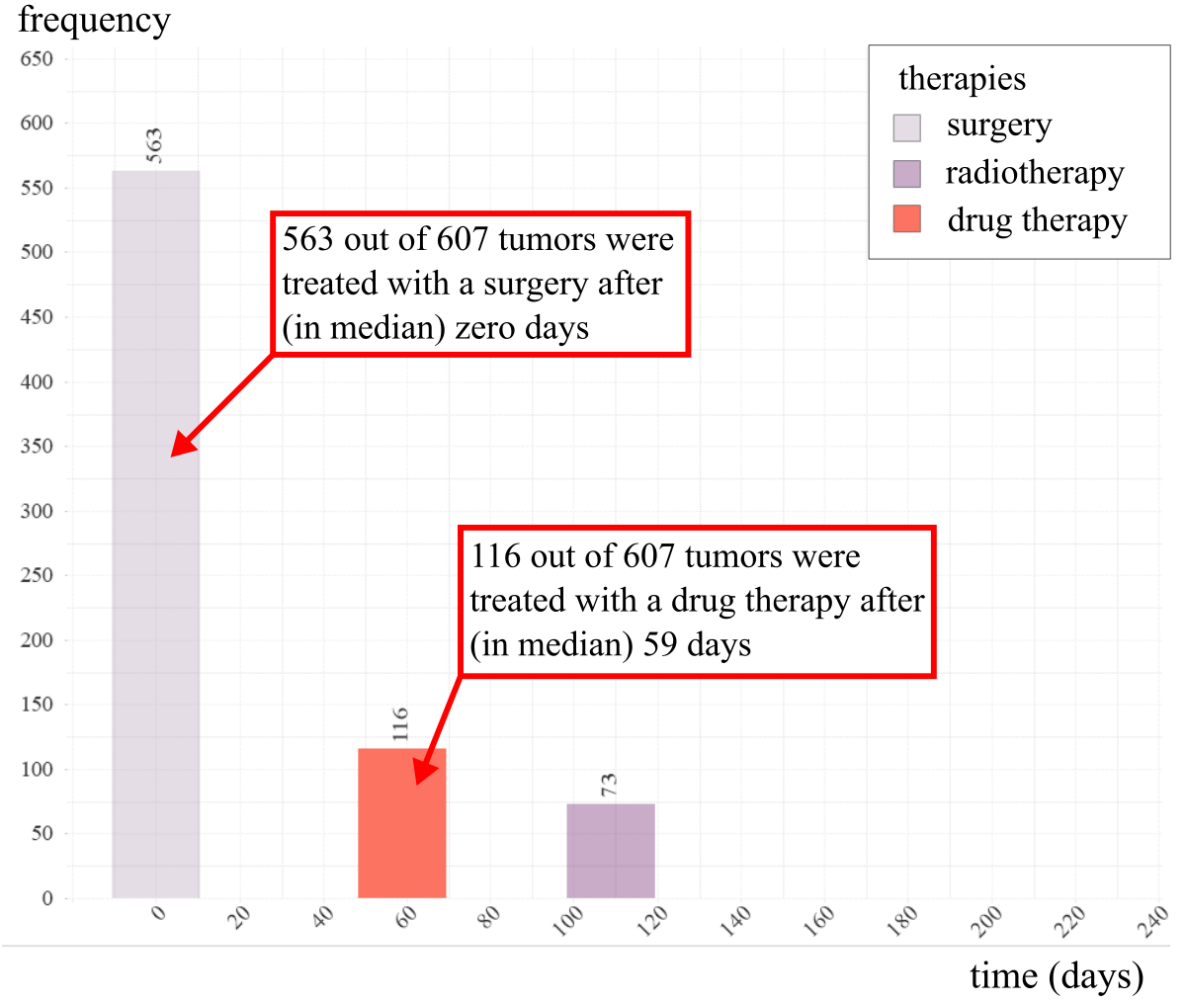
Median start at which a tumor of a chosen cohort has been treated with a specific therapy. The x-axis shows the time (in days), while the y-axis displays how many tumors have been treated in this cohort. In this example, for a cohort of 607 tumors, 563 have been treated with surgery (median after zero days), 116 with drug therapy (median after 59 days), and 73 with radiotherapy (median after 109 days).

Next, we created two more complex, integrated modules. The first module displays therapy timelines and the second focuses on survival. The survival module and its implementation were previously presented at the International Conference on Informatics, Management, and Technology in Healthcare 2019 [22]. In terms of visualizing the sequential flow of therapies, it is possible to display, on a time axis, the median start time after diagnosis of specific therapies (eg, first radiation or surgery) of a chosen patient cohort. This provides an overview of typical patterns, and is meant to create scientific interest, while providing general information about the chosen cohort and how the patients were treated. Figure 4 shows an example of such a pattern for a selected cohort, where most patients typically started out with a surgery, followed by a drug therapy in some cases (59 days), and concluded by radiotherapy (109 days). Similar to all contents within the system, this module reacts to any frontend selections (clicks) and changes the pattern according to the chosen cohort within seconds.

### Permission System

As mentioned above, when granting access to the platform, we had to conform to European as well as to local Bavarian laws of privacy protection [23,24]. First, data access in the initial release of this platform had to be restricted to administrators as well as local clinicians. Giving access to external scientists or partners is currently not planned, as full anonymization of data, which would be required in such a case, does not seem feasible [23]. Technically, we utilized QlikView’s capability to lock specific data field selections within a module on a per-user basis [25].

The Bavarian Law of Hospitals (Bayerisches Krankenhausgesetz (BayKRG) Art 27– Datenschutz (4)) served as the legal basis of our permission system [24]. This law states that physicians within a hospital are allowed to work with patient data (even in terms of research and without given consent) as long as they were involved in the process of care of that patient. According to the law, the physicians themselves are allowed to grant data access to other clinicians within the hospital as long as the data remain within the hospital. Hence, if a clinician asks for access to the platform, after signing the terms of agreement, we restrict their view to only patients formally in their care (or care unit). This was achieved by restricting the view based on filters within the data fields “organ (ICD-10)” and “treatment center.” As an example, a physician in the field of pulmonology will only be able to view data and cases pertaining to the patients who visited the lung center. This same physician will not be able to view liver tumors, for example, even though both entities exist within our dataset.

This system functions for clinicians working for organ-specific treatment centers (eg, a women’s hospital) or clinicians who are involved in the care of patients suffering from tumors of a specific organ. However, this system does not take into consideration clinicians working in interdisciplinary areas such as radiology. Therefore, we also included the possibility to restrict according to specific types of therapy (radiation, surgery, or drug therapy). Consequently, a radiologist with given permission would only be enabled to view data of patients who had indeed received radiotherapy and had been treated at the radiologist’s center. Hence, our system provides the physicians no additional information than they would normally be allowed to access. However, instead of having to sift through the information in all of the individual doctors’ reports, they are now able to directly access the aggregated data extracted from these reports. Hence, QlikView basically facilitates analysis, and helps with visualizing the cohort for which they are already responsible.

Such permission arrangements explain how the system has currently been rolled out and how it has been accepted by the privacy protection commissioner of the hospital. We here turn to describing how the permission system should be extended in the future. According to the Bavarian Law of Hospitals, clinicians are also allowed to share data (eg, for research purposes) within the hospital. This is more of a governance problem and has not yet been implemented within our current system. However, for the sake of scientific progress, clinicians interested in organ-specific data should be allowed to request permission to access relevant data even for cases in which they were not part of the original patient care. Representatives of the organ center would be members of a committee that could initially process such requests. The request will then be referred to a board consisting of the initial committee members along with organ center–independent members of the overreaching comprehensive cancer center. If all parties of the committee accept the request, extended data access will be granted. However, extended data access would only be given in a pseudonymized form, since full data access seems only reasonable for clinicians directly involved with a patient’s care.

The permission system, including the not-yet implemented extended data access, is organized via a permission table, which directly controls the contents that may be shown to a given user and within which timeframe. Table 1 provides an extract of our current permission table. In this example, the user “dnasseh” was given access to all patients of the lung tumor center, which all have a diagnosis code of C34. In contrast, the user “sopsch” would be allowed to see all radiated cases. As sopsch would be interested in liver cases as well, after a proposal, a committee would have granted them with extended permissions for liver-related data (C22) in a pseudonymized form (extended permissions are not yet implemented and are in a conceptual status).

**Table 1:** Example of a permission table for user access.

User	Diagnosis (ICD-10)^a^	Center	Radiation	Operation	System-therapy	Pseudo
dnasseh	C34	LTC^b^	*^c^	*	*	*
sopsch	*	*	STR^d^	*	*	*
sopsch	C22	*	*	*	*	PSD^e^

^a^ICD-10: International Statistical Classification of Diseases and Related Health Problems-10.

^b^LTC: lung tumor center.

^c^* represents a wildcard, meaning the user has full rights to view the contents within this column.

^d^STR: radiation.

^e^PSD: pseudonymized.

## Results

We established a platform that is accessible through the clinical intranet via a Web browser and does not require the installation of additional software at the end users’ sites. The data within the platform are updated daily, and provide preprocessed, compact visual access to the vast majority of the CREDOS contents. The platform can only be accessed after a single user license has been acquired. Based on this, the data from cohorts that the users can view are limited by a permission system, which was developed in parallel to the technical implementation. A nonlegal contract describes the rules for licensing and accesses to the platform. Before each login, a disclaimer has to be ticked (see Multimedia Appendix 1).

We structured the contents into six main categories, five of which (first assessment, patient baseline data, therapies, progression, survival) have been included in the first rollout version. To facilitate understanding of this complex system, Multimedia Appendix 1 shows screenshots (with labels translating the contents) of the layout for all of these categories and its included modules. Due to concerns of privacy protection, we blurred sensitive information within these screenshots.

As it is hard to describe the dynamic analytical possibilities of the platform, we provide a 15-minute-long commented video in Multimedia Appendix 2, displaying the real-time assembly of arbitrary data cohorts, dynamic interconnectivity between any data tables or data objects, as well as possibilities of the visual modules and its interaction with the other objects of the platform. This video was compiled to give an accurate impression about what the platform is capable to do.

## Discussion

### Feedback on the System

One of the major benefits of the MOCCA system is transparency. Until creation of the framework, clinicians themselves did not have the option to directly access aggregated CREDOS data. Instead, they had to assess individual patient records or send a request for help to the local information technology team. This process restricts interest in the data. Hence, for most physicians, the CREDOS dataset is comparable to a black box that is primarily used by documentation clerks and the information technology department. Thus, most clinicians were not aware of the rich contents of the database. The system was rolled out and evaluated at one of the partnering sites (CCCM-LMU). After providing the doctors access to the platform within our prerollout phase, we received extensive feedback about the contents of our database.

This feedback reflected the high quality of the data, along with areas of further improvement for some aspects. Along these lines, due to the richness of charts and graphs, it is very easy to spot incorrectly documented information. Since a doctor can browse through the data, they will quickly realize if any data are missing or not documented in a correct manner. As an example, some wrongly documented dates could easily be spotted as they showed up within the therapy time chart as a negative time value on the x-axis. Spotting inconsistencies is of high relevance, since high data quality is one of the requirements for clinical research and is a precondition for clinical trials or network activities such as those of the national Network Genomic Medicine for lung cancer, which locally relies on correct, complete, and valid tumor documentation data [26]. For example, we received feedback that the method of documenting our radiotherapies was not fully in accordance to standards set for radiologists. Hence, we were able to address these issues within our database. Since the initial implementation of the MOCCA system, we have received valuable and extensive feedback about our data contents, not only from information technology or tumor document specialists but also directly from the physicians.

Improving the data quality itself is only one of the purposes of MOCCA; it can also support the control of processes of routine care and identify potential risks. Regarding this aspect of quality control, clinicians did not have the right tools to directly and quickly assess whether quality of care and associated processes were acceptable. As the centers are certified once a year by OnkoZert [17], they have to prepare and display specific key performance indicators such as the number of patient cases introduced to the tumor boards. Since substantial attention is focused on this event, quality problems that occur between these events might be identified too late and pose problems when attempting to overcome these shortcomings. Naturally, controlling should not be, and is not, limited to the annual certification events. The different organ centers regularly request information about the CREDOS contents (eg, amount of cases with a specific therapy). This information can be supplied by the information technology staff or documentation clerks. However, with more classical statistics tools, scripts would have to be written and altered for every analysis request. Hence, a capable business intelligence platform such as the MOCCA system provides a more direct approach, while avoiding unnecessary waiting times and communicative misunderstandings. The process is further supported by the in-memory technology, which allows real-time analyses of arbitrary data cohorts [11] and automatic daily data updates to ensure that the information available to interested physicians is always current.

### Strengths and Limitations

When critically assessed, a potential shortcoming of the system is misinterpretation caused by visual inspection of data, without considering the influences of confounding factors, sample size, and sample bias [27]. For example, when viewing the documented total occurrences of cancer cases of the CCC-LMU (Figure 2), it can be noted that the number of tumor cases per year has been rising. However, this does not mean that the occurrence of tumor cases is rising on a population level, but rather that the completeness of documentation or the frequency of patients seeking treatment for these types of tumors has increased throughout the years. As long as it is understood that MOCCA is not an epidemiological overview, but rather a local view on the present documented cancer data, we believe that a system like MOCCA can support analysis of local data and create exciting research opportunities.

Although our system presents and analyzes data in graphic detail, we recommend that the results and conclusions mined from our system should always be examined with the support of statisticians, medical computer scientists, and in comparison to larger datasets (eg, epidemiological registries) [28]. In fact, in line with Bauer et al [29], we included a disclaimer within the system that warns of misinterpretation and indicates the necessity to consult information technology or statistical professionals [29] (see Multimedia Appendix 1). Additionally, the system offers tooltips, text fields, and popups explaining most of the contents, and even provides direct references when using formulas, mostly within the survival module [22]. Since precautionary methods might be ignored, we set up another security measure in which we only hand out licenses after a direct tutorial session, discussing not only the benefits but also the limitations and dangers of the system.

**Figure 5:**
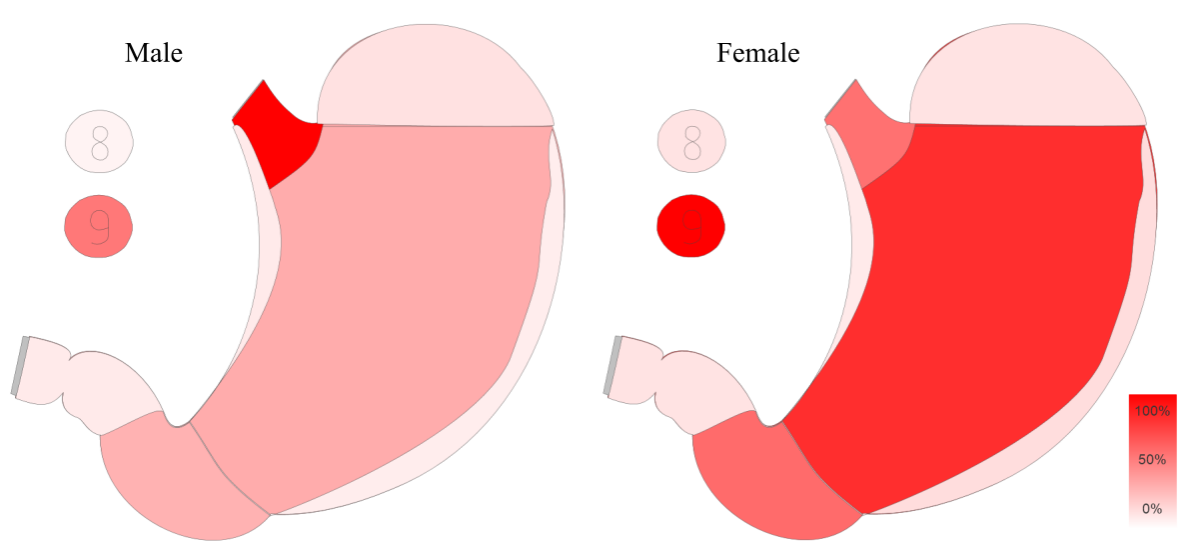
Comparison of two different cohorts within the organ map module. Different patterns of occurrence for female and male stomach cancer cases are evident. The color saturation is linear to the occurrence, with 100% saturation being the most affected segment.

In addition to the means and methods with which this system can support research, the system can also quickly and easily provide numbers for formulating scientific proposals. Furthermore, it can be used to discover as-yet-unknown information, also referred to as knowledge or data discovery, that can contribute to creating research ideas by quickly browsing through the data (hypothesis generation) [14,30]. For example, the organ map displays the relative occurrence of a tumor type in a specific location, which was treated at the center. As we are able to alter the data cohort arbitrarily, we can switch from male patients to female patients in real time, which changes the coloration of the organ map (Figure 5).

The benefits of such data visualization and business intelligence have been previously discussed in multiple contexts [31-33]. However, this is one more example for the effectiveness of such systems, which is based on the balance shift between perception and cognition [34]. In this way, we can quickly realize the most significant changes within both graphics. We provide an example in Figure 5, in which it appears easy to identify that the cardia (opening of the stomach) is more affected among males than among females. Such a finding can result in the formulation of new research ideas and support hypothesis generation [30]. In this context, the second big limitation within our system is governance. Physicians employed at a cooperating center (at the same site) should not be able to examine data mined from patients of another center. This is particularly tricky when granting rights to interdisciplinary physicians like radiologists, whose patients would have also been treated at other organ centers. Hence, publications using data derived from multiple sites can result in conflicts. To address and deflate this concern, it is important to point out that MOCCA only facilitates an aggregated data view, but will not give more information than physicians can already find when looking into the individual digital patient files of their aggregated system for whom they were granted access according to hospital policy. Consequently, all information within CREDOS could also be found within a well-made shadow database. The difference is that the CCCM at both sites comprises staff that are professional documentation specialists, whereas associated centers do not necessarily have these resources. Often, these shadow databases are created with the support of students who lack the experience, education, and training for creating a clean dataset. However, this should not be generalized, and having individual databases is important for research, especially since these databases might contain additional information not found within CREDOS. Nevertheless, a business intelligence tool such as MOCCA can only improve these already existing systems and possibly make them, or large parts of them, redundant, resulting in a reduction of necessary capacities as well as an increase of professionalism in terms of documentation, data quality, and transparency. In this regard, we received positive feedback in our test phase when some of the physicians realized that one of their databases, which was created parallel to our system and is also used for annual audits, might be superfluous since all data needed can be supplied by the larger CREDOS system. This could have a direct impact and release bound capacities, reducing the need for each individual center to create their own systems.

In terms of measurable benefit, as the system was only recently released, the physicians have not yet utilized it for extensive research projects. Nevertheless, it serves as a quick help for everyday routine requests and is already an essential part of our annual audits. In general, we can summarize that the feedback of the prerollout was primarily positive, and it is safe to say that it generated interest in, and led to voluntary confrontation of doctors with the data, which in turn opened the door to translational interaction, improved data quality, and possibly research. An objective measurement about the benefit of the tool (eg, based on surveys, citations, or login frequencies) is planned for the future.

### Conclusions and Prospects

As for future perspectives, due to the success and mostly positive feedback of our testing rollout, we aim to launch MOCCA at our partnering site (CCCM-Technical University Munich). In terms of contents, we would like to add further standard statistics and integrate some additional modules such as a single patient view. Moreover, these innovative data management techniques, and the handling of permissions and data governance, which is known as a major hurdle for many health-related projects, should serve as an inspiration for similar projects at other sites both nationally and internationally [35].

## Appendix

Multimedia Appendix 1 Overview over all components (with translations) of the given MOCCA system.

Multimedia Appendix 2 Video summarizing the capabilities of the platform.

## Abbreviations

CCCM: Comprehensive Cancer Center Munich
CREDOS: Cancer Retrieval Evaluation and Documentation System
ICIMTH: International Conference on Informatics, Management and Technology in Healthcare
LDAP: lightweight directory access protocol
LMU: Ludwig-Maximilians University, Munich
LTC: Lung Tumor Center
MOCCA: Munich Online Comprehensive Cancer Analytics
PSD: Pseudonymized
STR: Radiation
SVG: scalable vector graphics
