# Supplementary material for: Optimizing the Analytical Value of Oncology-Related Data Based on an In-Memory Analysis Layer: Development and Assessment of the Munich Online Comprehensive Cancer Analysis Platform
Source: J Med Internet Res. 2020 Apr 17;22(4):e16533. doi: 10.2196/16533 (PMC7195671; doi:10.2196/16533)
Supplement: Multimedia Appendix 1 [file jmir_v22i4e16533_app1.pdf]

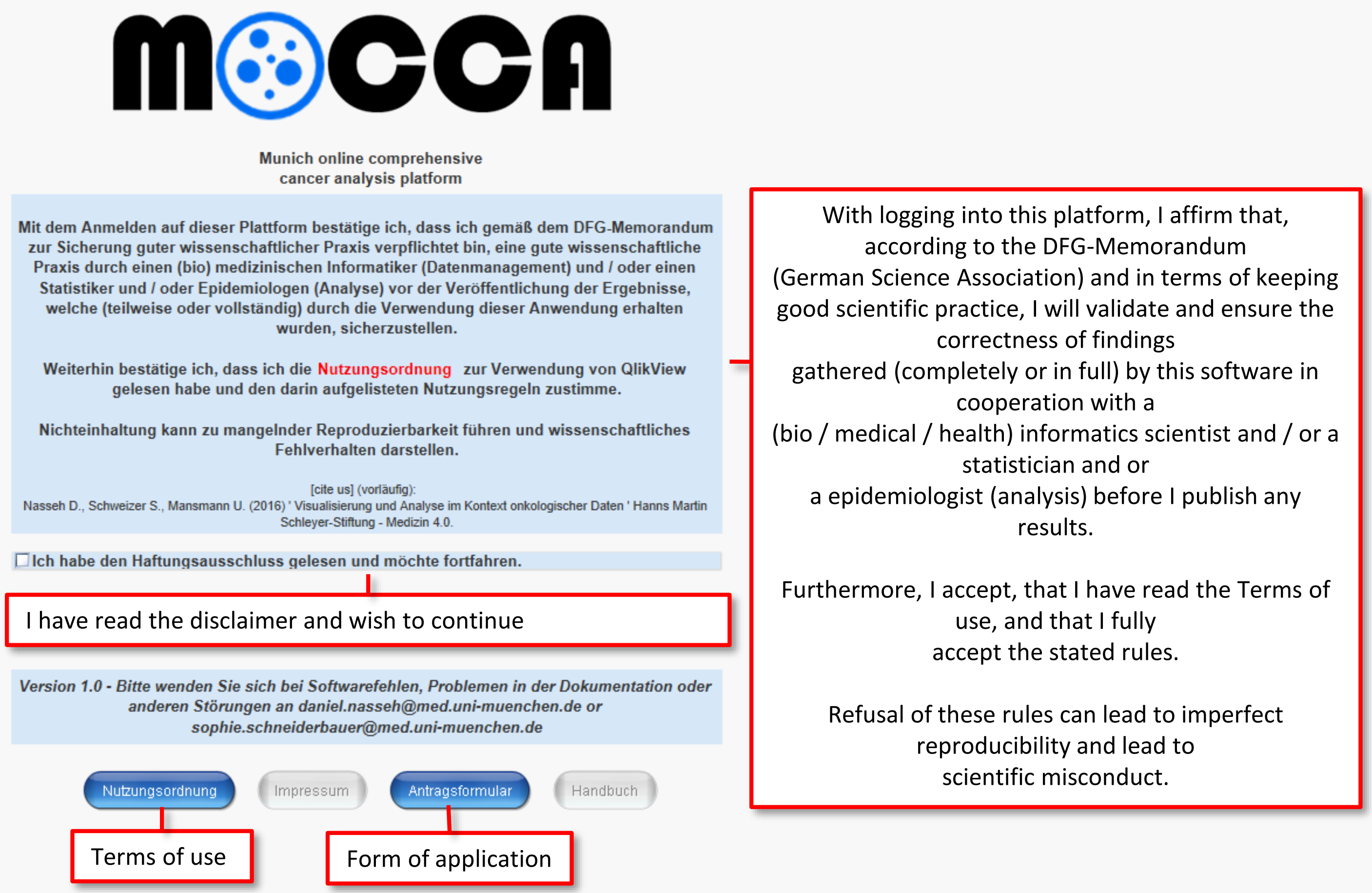
Munich online comprehensive cancer analysis platform
Mit dem Anmelden auf dieser Plattform bestätige ich, dass ich gemäß dem DFG-Memorandum zur Sicherung guter wissenschaftlicher Praxis verpflichtet bin, eine gute wissenschaftliche Praxis durch einen (bio) medizinischen Informatiker (Datenmanagement) und / oder einen Statistiker und / oder Epidemiologen (Analyse) vor der Veröffentlichung der Ergebnisse, welche (teilweise oder vollständig) durch die Verwendung dieser Anwendung erhalten wurden, sicherzustellen.
Weiterhin bestätige ich, dass ich die Nutzungsordnung zur Verwendung von QlikView gelesen habe und den darin aufgelisteten Nutzungsregeln zustimme.
Nichteinhaltung kann zu mangelnder Reproduzierbarkeit führen und wissenschaftliches Fehlverhalten darstellen.
[cite us] (vorläufig):
Nasseh D., Schweizer S., Mansmann U. (2016) ' Visualisierung und Analyse im Kontext onkologischer Daten ' Hanns Martin Schleyer-Stiftung - Medizin 4.0.
Ich habe den Haftungsausschluss gelesen und möchte fortfahren.
I have read the disclaimer and wish to continue
Version 1.0 - Bitte wenden Sie sich bei Softwarefehlen, Problemen in der Dokumentation oder anderen Störungen an daniel.nasseh@med.uni-muenchen.de oder sophie.schneiderbauer@med.uni-muenchen.de
Nutzungsordnung
Impressum
Antragsformular
Handbuch
Terms of use
Form of application
With logging into this platform, I affirm that, according to the DFG-Memorandum (German Science Association) and in terms of keeping good scientific practice, I will validate and ensure the correctness of findings gathered (completely or in full) by this software in cooperation with a (bio / medical / health) informatics scientist and / or a statistician and or a epidemiologist (analysis) before I publish any results.
Furthermore, I accept, that I have read the Terms of use, and that I fully accept the stated rules.
Refusal of these rules can lead to imperfect reproducibility and lead to scientific misconduct.

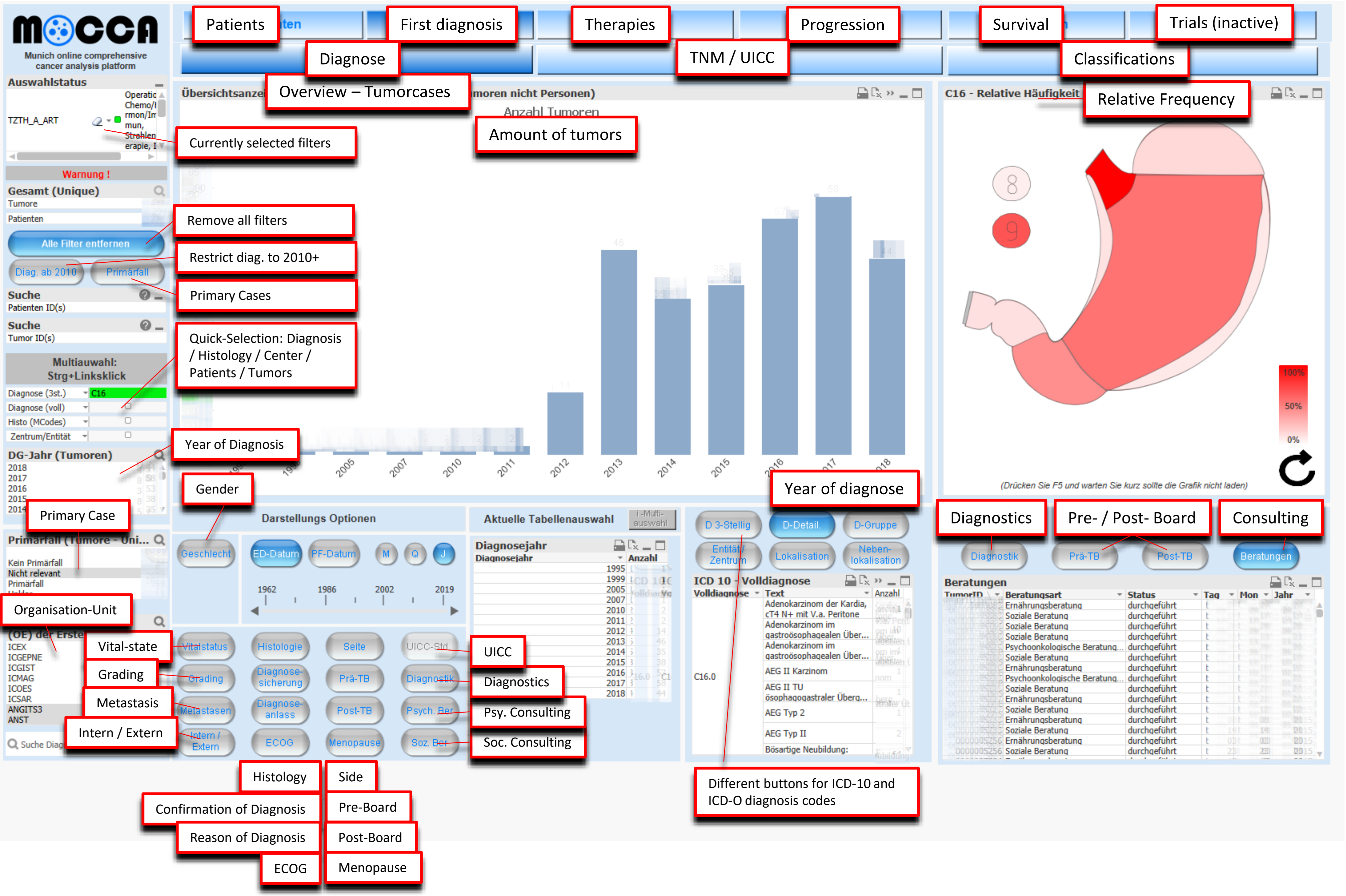

Patients
First diagnosis
Therapies
Progression
Survival
Trials (inactive)
Diagnose
TNM / UICC
Classifications
Overview – Tumorcases
Relative Frequency
Currently selected filters
Amount of tumors
Remove all filters
Restrict diag. to 2010+
Primary Cases
Quick-Selection: Diagnosis / Histology / Center / Patients / Tumors
Year of Diagnosis
Gender
Primary Case
Year of diagnose
Diagnostics
Pre- / Post- Board
Consulting
Organisation-Unit
Vital-state
Grading
Metastasis
Intern / Extern
UICC
Diagnostics
Psy. Consulting
Soc. Consulting
Histology
Side
Confirmation of Diagnosis
Pre-Board
Reason of Diagnosis
Post-Board
ECOG
Menopause
Different buttons for ICD-10 and ICD-O diagnosis codes

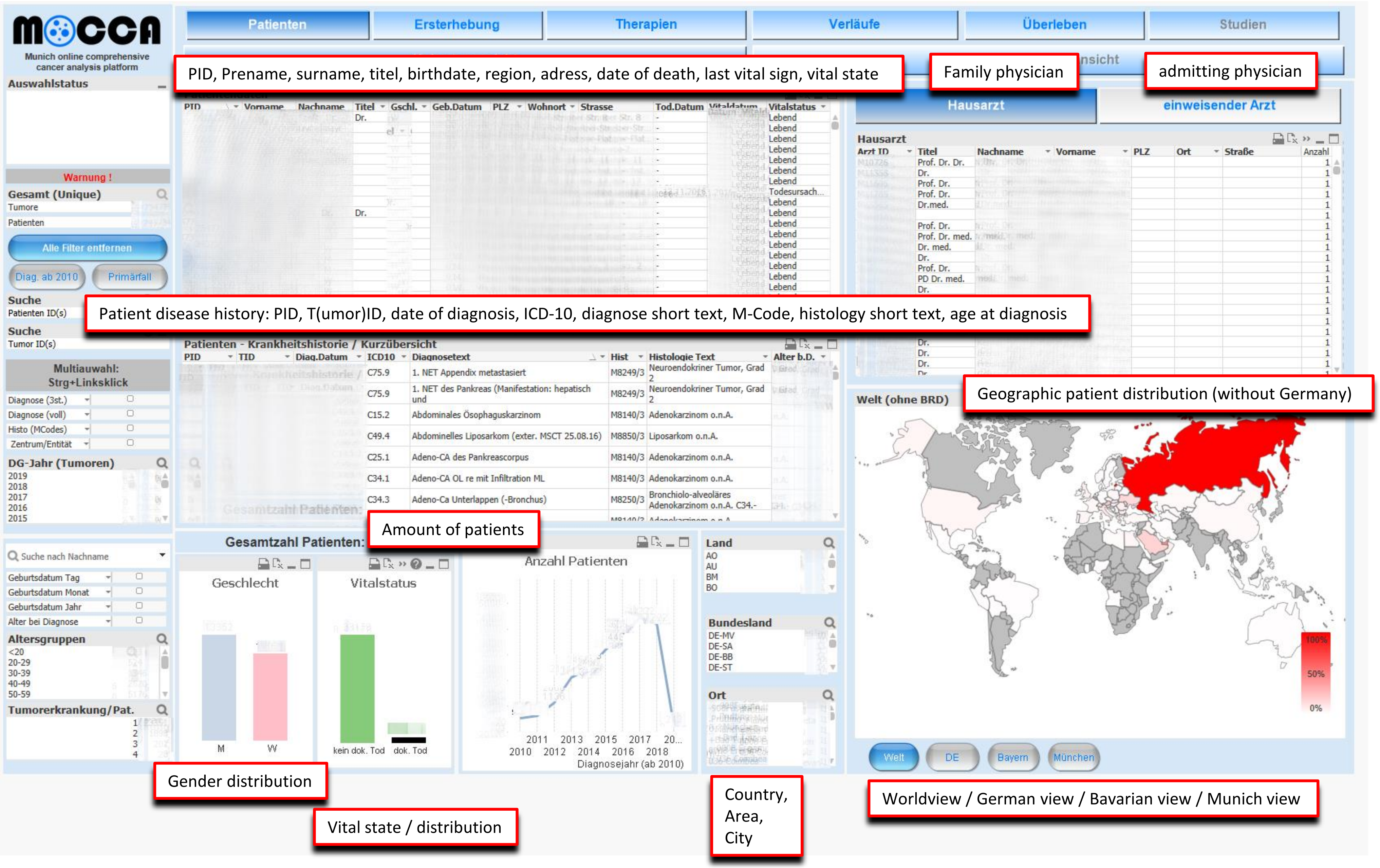

MOCCA
Munich online comprehensive cancer analysis platform
Patienten
Ersterhebung
Therapien
Verläufe
Überleben
Studien
PID, Prename, surname, titel, birthdate, region, adress, date of death, last vital sign, vital state
Family physician
admitting physician
Hausarzt
einweisender Arzt
Auswahlstatus
Warnung !
Gesamt (Unique)
Tumore
Patienten
Alle Filter entfernen
Diag. ab 2010
Primärfall
Suche
Patienten ID(s)
Suche
Tumor ID(s)
Patient disease history: PID, T(umor)ID, date of diagnosis, ICD-10, diagnose short text, M-Code, histology short text, age at diagnosis
Patienten - Krankheitshistorie / Kurzübersicht
Multiauswahl: Strg+Linksklick
DG-Jahr (Tumoren)
Amount of patients
Gesamtzahl Patienten:
Geschlecht
Vitalstatus
Anzahl Patienten
Diagnosejahr (ab 2010)
Land
Bundesland
Ort
Welt (ohne BRD)
Geographic patient distribution (without Germany)
Welt
DE
Bayern
München
Altersgruppen
Tumorerkrankung/Pat.
Gender distribution
Vital state / distribution
Country, Area, City
Worldview / German view / Bavarian view / Munich view

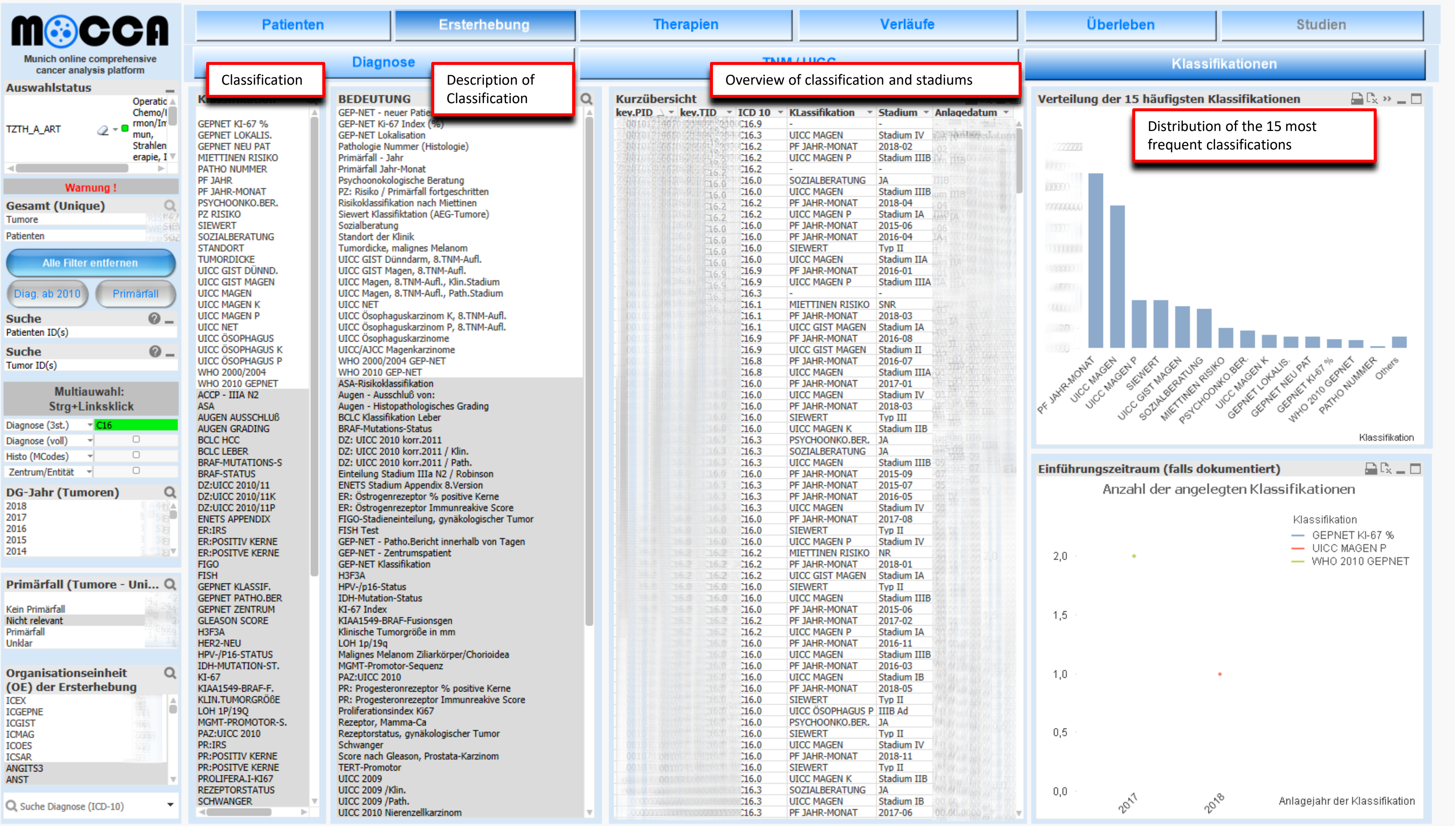
MOCCA
Munich online comprehensive cancer analysis platform
Patienten
Ersterhebung
Therapien
Verläufe
Überleben
Studien
Diagnose
TNM / UICC
Klassifikationen
Classification
Description of Classification
Overview of classification and stadiums
Distribution of the 15 most frequent classifications
Auswahlstatus
Warnung !
Gesamt (Unique)
Tumore
Patienten
Alle Filter entfernen
Diag. ab 2010
Primärfall
Suche
Patienten ID(s)
Tumor ID(s)
Multiauswahl: Strg+Linksklick
Diagnose (3st.)
C16
Diagnose (voll)
Histo (MCodes)
Zentrum/Entität
DG-Jahr (Tumoren)
Primärfall (Tumore - Uni...
Kein Primärfall
Nicht relevant
Primärfall
Unklar
Organisationseinheit (OE) der Ersterhebung
Suche Diagnose (ICD-10)
BEDEUTUNG
Kurzübersicht
key.PID
key.TID
ICD 10
KLassifikation
Stadium
Anlagedatum
Verteilung der 15 häufigsten Klassifikationen
PF JAHR-MONAT
UICC MAGEN
UICC MAGEN P
SIEWERT
UICC GIST MAGEN
SOZIALBERATUNG
MIETTINEN RISIKO
PSYCHOONKO.BER.
UICC MAGEN K
GEPNET LOKALIS.
GEPNET NEU PAT
GEPNET KI-67 %
WHO 2010 GEPNET
PATHO NUMMER
Others
Klassifikation
Einführungszeitraum (falls dokumentiert)
Anzahl der angelegten Klassifikationen
Klassifikation
GEPNET KI-67 %
UICC MAGEN P
WHO 2010 GEPNET
2,0
1,5
1,0
0,5
0,0
2017
2018
Anlagejahr der Klassifikation

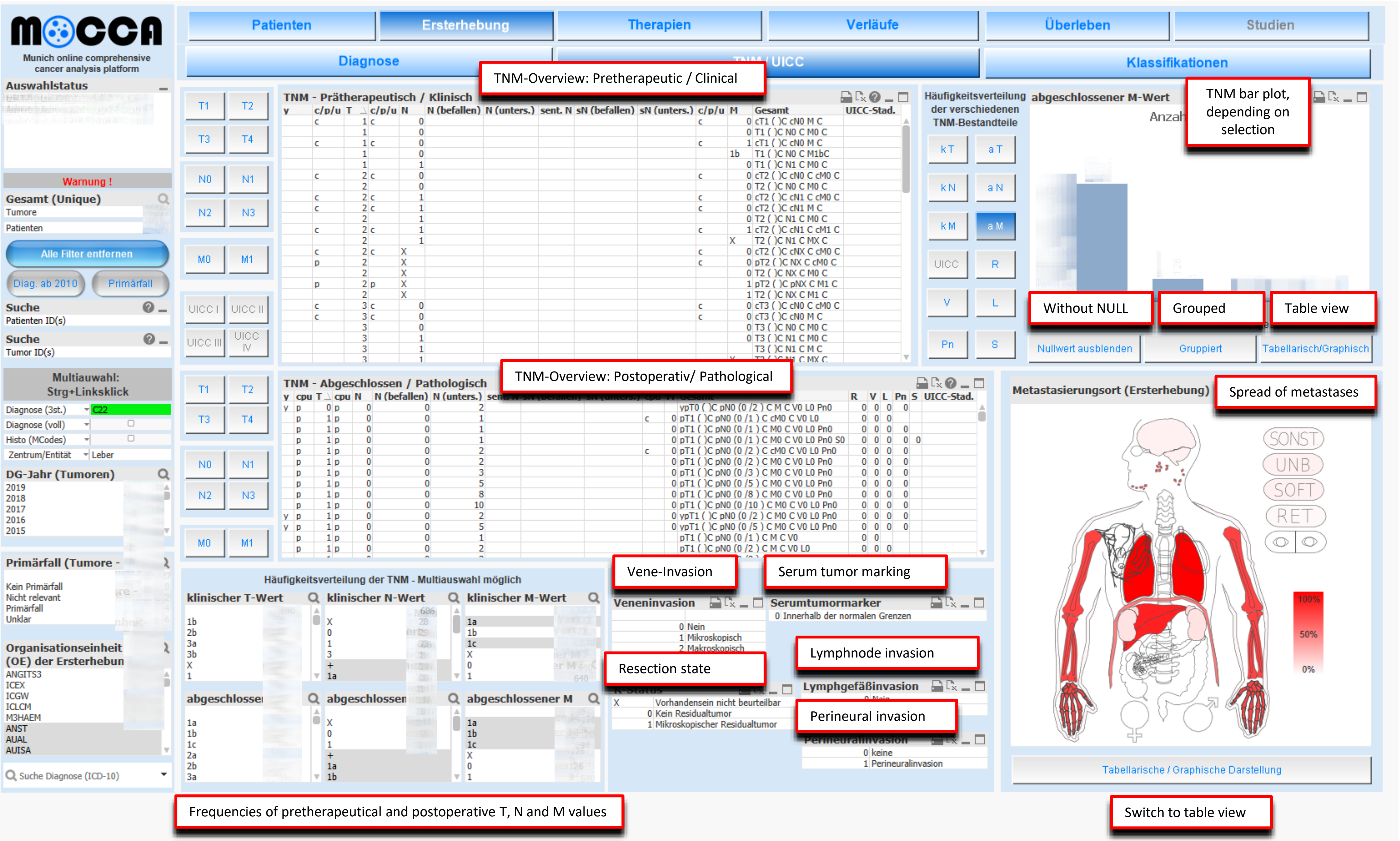

Munich online comprehensive cancer analysis platform
Patienten
Ersterhebung
Therapien
Verläufe
Überleben
Studien
Diagnose
TNM / UICC
Klassifikationen
TNM-Overview: Pretherapeutic / Clinical
TNM - Prätherapeutisch / Klinisch
TNM bar plot, depending on selection
Häufigkeitsverteilung der verschiedenen TNM-Bestandteile
abgeschlossener M-Wert
Without NULL
Grouped
Table view
Nullwert ausblenden
Gruppiert
Tabellarisch/Graphisch
TNM-Overview: Postoperativ/ Pathological
TNM - Abgeschlossen / Pathologisch
Metastasierungsort (Ersterhebung)
Spread of metastases
Vene-Invasion
Serum tumor marking
Veneninvasion
Serumtumormarker
Lymphnode invasion
Resection state
Lymphgefäßinvasion
Perineural invasion
Perineuralinvasion
Häufigkeitsverteilung der TNM - Multiauswahl möglich
klinischer T-Wert
klinischer N-Wert
klinischer M-Wert
abgeschlossener M
Tabellarische / Graphische Darstellung
Frequencies of pretherapeutical and postoperative T, N and M values
Switch to table view
Auswahlstatus
Warnung !
Gesamt (Unique)
Alle Filter entfernen
Diag. ab 2010
Primärfall
Suche
Multiauswahl: Strg+Linksklick
DG-Jahr (Tumoren)
Organisationseinheit (OE) der Ersterhebun
Suche Diagnose (ICD-10)

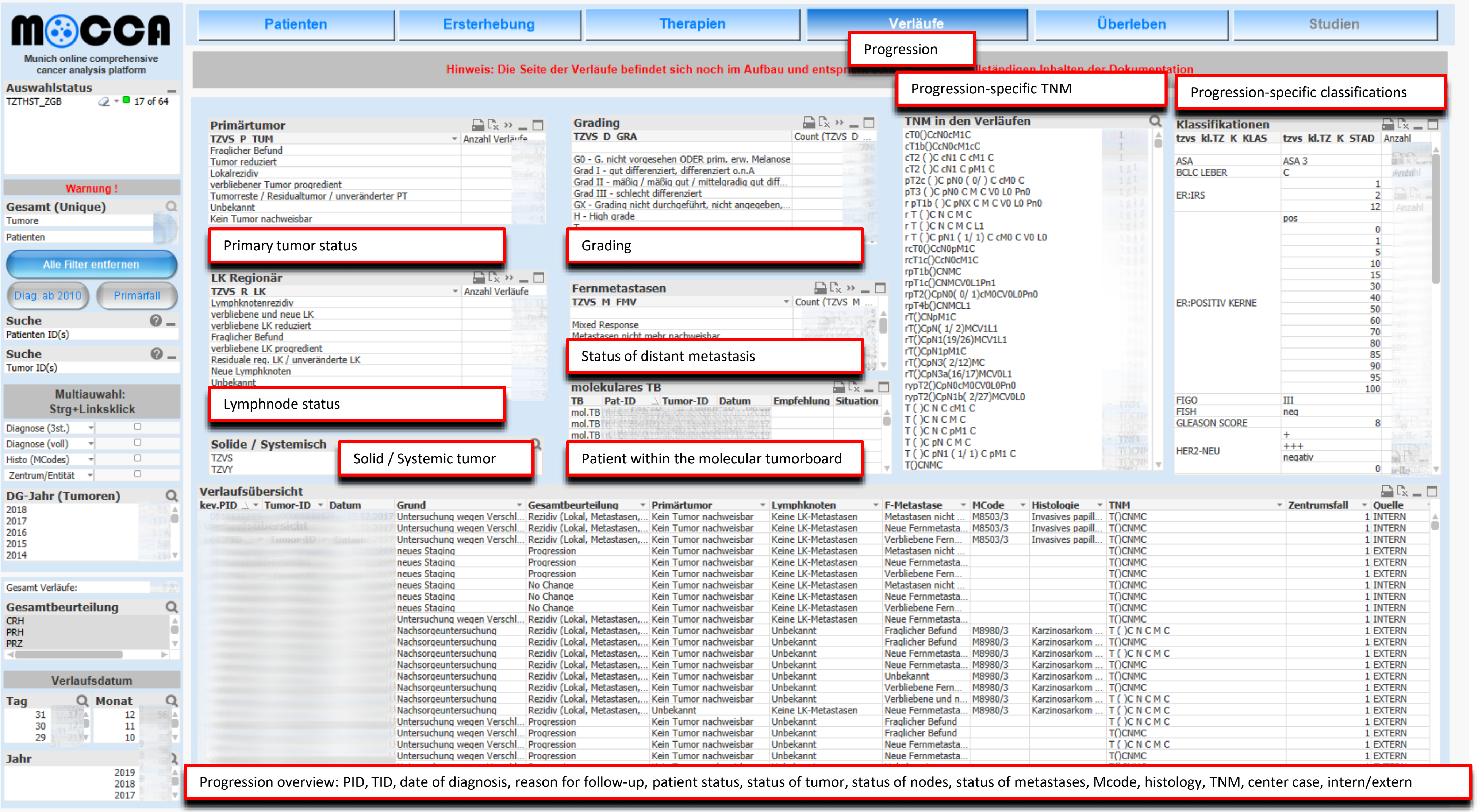

Munich online comprehensive cancer analysis platform
Patienten
Ersterhebung
Therapien
Verläufe
Überleben
Studien
Progression
Hinweis: Die Seite der Verläufe befindet sich noch im Aufbau und entspr
Progression-specific TNM
Progression-specific classifications
Primärtumor
Grading
TNM in den Verläufen
Klassifikationen
Primary tumor status
Grading
LK Regionär
Fernmetastasen
Status of distant metastasis
molekulares TB
Lymphnode status
Solide / Systemisch
Solid / Systemic tumor
Patient within the molecular tumorboard
Verlaufsübersicht
Progression overview: PID, TID, date of diagnosis, reason for follow-up, patient status, status of tumor, status of nodes, status of metastases, Mcode, histology, TNM, center case, intern/extern

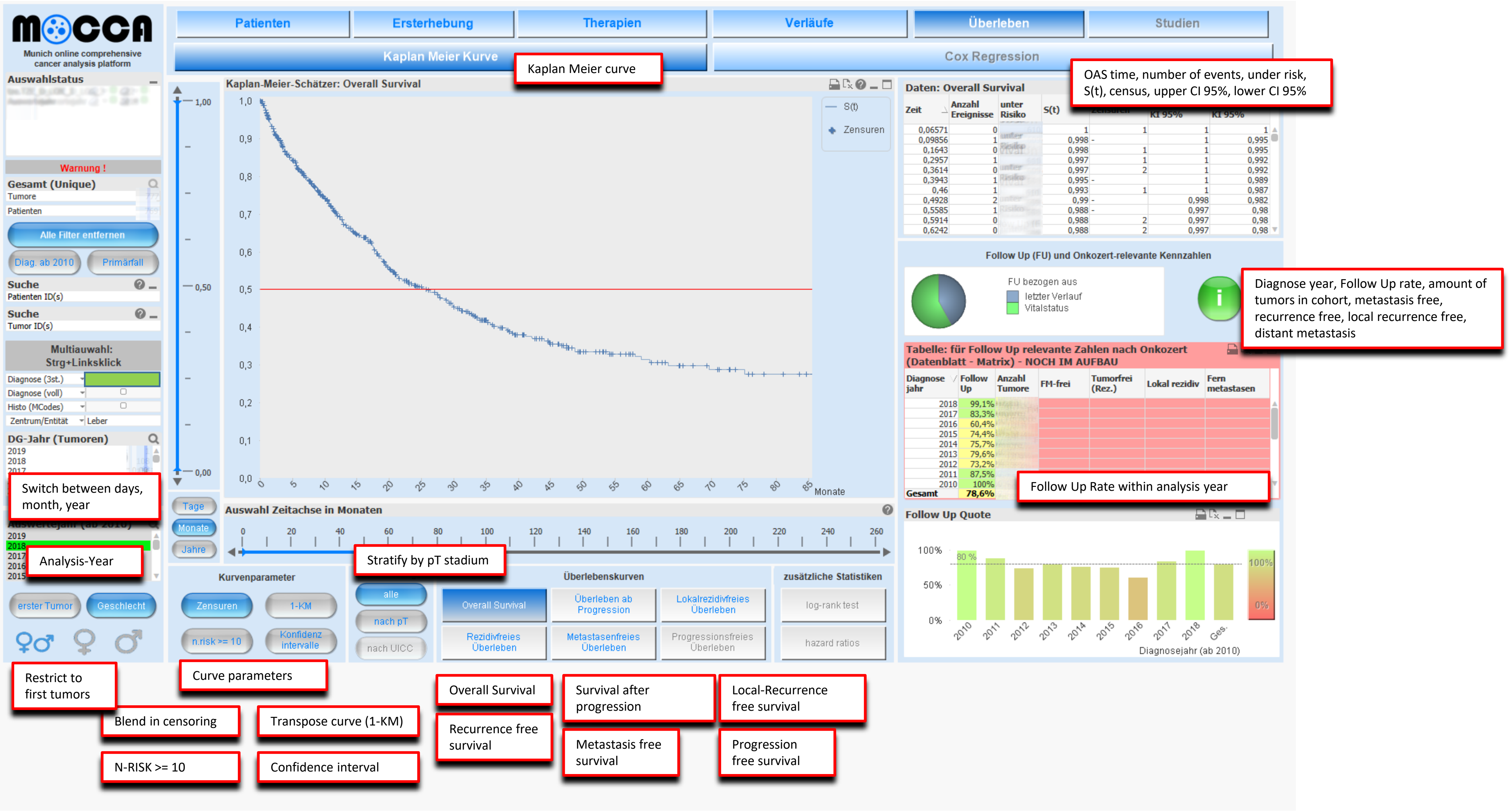

Munich online comprehensive cancer analysis platform
Patienten
Ersterhebung
Therapien
Verläufe
Überleben
Studien
Kaplan Meier Kurve
Cox Regression
Kaplan Meier curve
OAS time, number of events, under risk, S(t), census, upper CI 95%, lower CI 95%
Kaplan-Meier-Schätzer: Overall Survival
Daten: Overall Survival
Follow Up (FU) und Onkozert-relevante Kennzahlen
Diagnose year, Follow Up rate, amount of tumors in cohort, metastasis free, recurrence free, local recurrence free, distant metastasis
Tabelle: für Follow Up relevante Zahlen nach Onkozert (Datenblatt - Matrix) - NOCH IM AUFBAU
Follow Up Rate within analysis year
Follow Up Quote
Switch between days, month, year
Analysis-Year
Auswahl Zeitachse in Monaten
Stratify by pT stadium
Kurvenparameter
Überlebenskurven
zusätzliche Statistiken
Restrict to first tumors
Curve parameters
Blend in censoring
Transpose curve (1-KM)
N-RISK >= 10
Confidence interval
Overall Survival
Recurrence free survival
Survival after progression
Metastasis free survival
Local-Recurrence free survival
Progression free survival

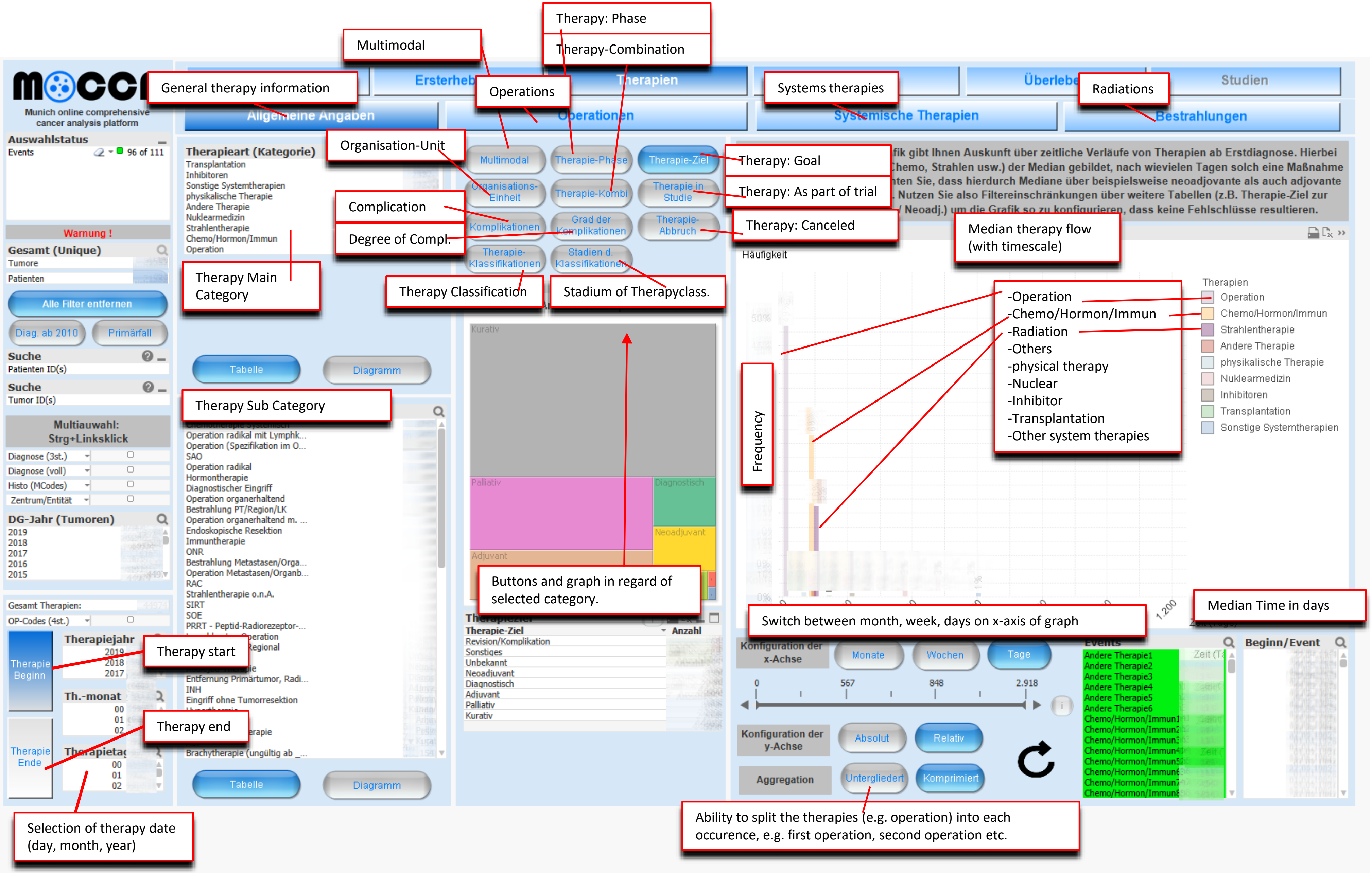

Therapy: Phase
Therapy-Combination
Multimodal
General therapy information
Operations
Systems therapies
Radiations
Organisation-Unit
Therapy: Goal
Therapy: As part of trial
Complication
Degree of Compl.
Therapy: Canceled
Median therapy flow (with timescale)
Therapy Main Category
Therapy Classification
Stadium of Therapyclass.
-Operation
-Chemo/Hormon/Immun
-Radiation
-Others
-physical therapy
-Nuclear
-Inhibitor
-Transplantation
-Other system therapies
Frequency
Therapy Sub Category
Buttons and graph in regard of selected category.
Median Time in days
Switch between month, week, days on x-axis of graph
Therapy start
Therapy end
Selection of therapy date (day, month, year)
Ability to split the therapies (e.g. operation) into each occurence, e.g. first operation, second operation etc.
Munich online comprehensive cancer analysis platform
Ersterheb
Therapien
Überlebe
Studien
Allgemeine Angaben
Operationen
Systemische Therapien
Bestrahlungen
Multimodal
Therapie-Phase
Therapie-Ziel
Organisations-Einheit
Therapie-Kombi
Therapie in Studie
Komplikationen
Grad der Komplikationen
Therapie-Abbruch
Therapie-Klassifikationen
Stadien d. Klassifikationen
Therapieart (Kategorie)
Transplantation
Inhibitoren
Sonstige Systemtherapien
physikalische Therapie
Andere Therapie
Nuklearmedizin
Strahlentherapie
Chemo/Hormon/Immun
Operation
Tabelle
Diagramm
Kurativ
Palliativ
Diagnostisch
Neoadjuvant
Adjuvant
Häufigkeit
Therapien
Operation
Chemo/Hormon/Immun
Strahlentherapie
Andere Therapie
physikalische Therapie
Nuklearmedizin
Inhibitoren
Transplantation
Sonstige Systemtherapien
Therapie-Ziel
Anzahl
Revision/Komplikation
Sonstiges
Unbekannt
Neoadjuvant
Diagnostisch
Adjuvant
Palliativ
Kurativ
Konfiguration der x-Achse
Monate
Wochen
Tage
Konfiguration der y-Achse
Absolut
Relativ
Aggregation
Untergliedert
Komprimiert
Beginn/Event
Therapiejahr
Th.-monat
Therapietag
Therapie Beginn
Therapie Ende

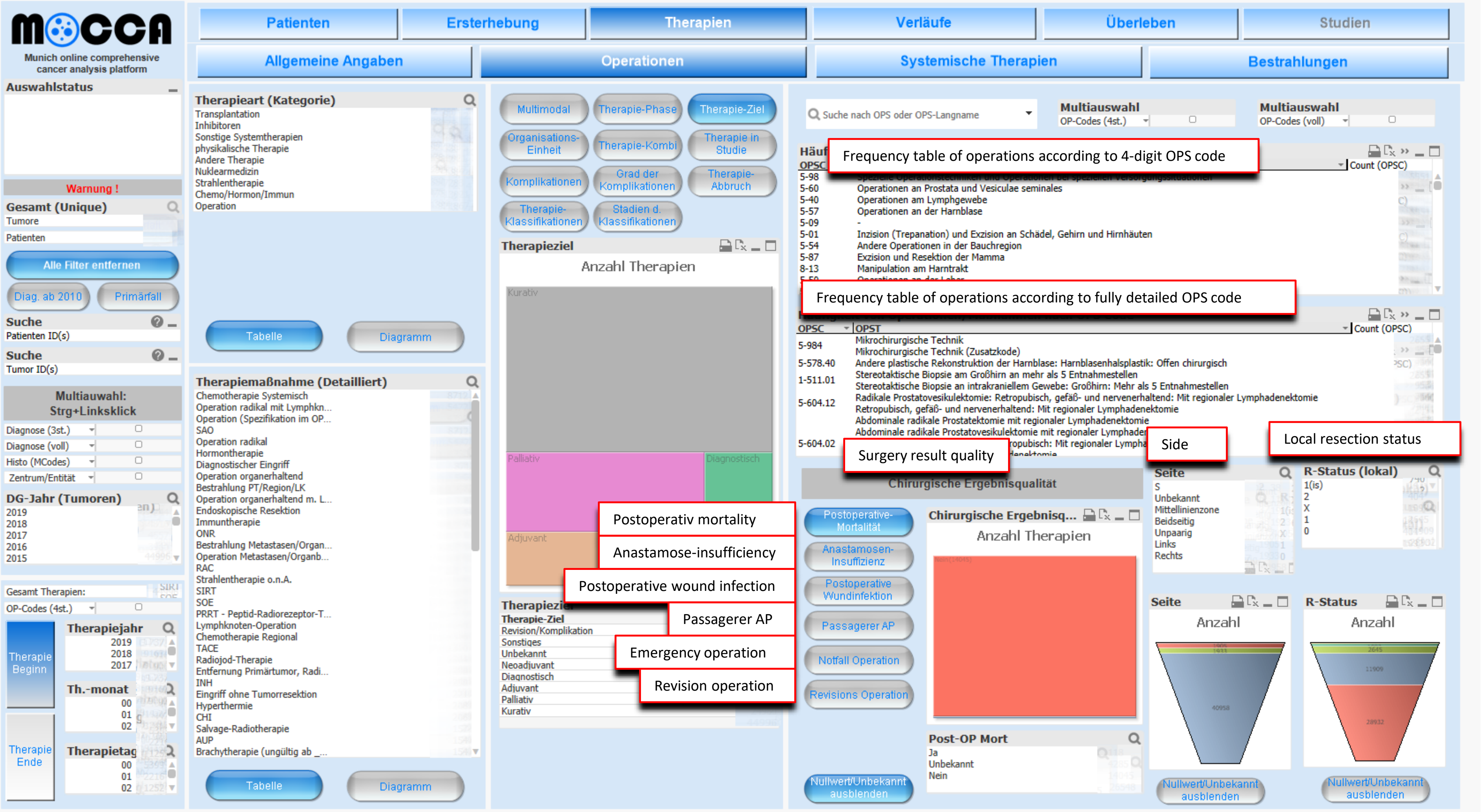

Munich online comprehensive cancer analysis platform
Patienten
Ersterhebung
Therapien
Verläufe
Überleben
Studien
Allgemeine Angaben
Operationen
Systemische Therapien
Bestrahlungen
Auswahlstatus
Warnung !
Gesamt (Unique)
Tumore
Patienten
Alle Filter entfernen
Diag. ab 2010
Primärfall
Suche
Patienten ID(s)
Suche
Tumor ID(s)
Multiauwahl: Strg+Linksklick
Diagnose (3st.)
Diagnose (voll)
Histo (MCodes)
Zentrum/Entität
DG-Jahr (Tumoren)
2019
2018
2017
2016
2015
Gesamt Therapien:
OP-Codes (4st.)
Therapie Beginn
Therapie Ende
Therapiejahr
2019
2018
2017
Th.-monat
00
01
02
Therapietag
00
01
02
Therapieart (Kategorie)
Transplantation
Inhibitoren
Sonstige Systemtherapien
physikalische Therapie
Andere Therapie
Nuklearmedizin
Strahlentherapie
Chemo/Hormon/Immun
Operation
Tabelle
Diagramm
Therapiemaßnahme (Detailliert)
Chemotherapie Systemisch
Operation radikal mit Lymphkn...
Operation (Spezifikation im OP...
SAO
Operation radikal
Hormontherapie
Diagnostischer Eingriff
Operation organerhaltend
Bestrahlung PT/Region/LK
Operation organerhaltend m. L...
Endoskopische Resektion
Immuntherapie
ONR
Bestrahlung Metastasen/Organ...
Operation Metastasen/Organb...
RAC
Strahlentherapie o.n.A.
SIRT
SOE
PRRT - Peptid-Radiorezeptor-T...
Lymphknoten-Operation
Chemotherapie Regional
TACE
Radiojod-Therapie
Entfernung Primärtumor, Radi...
INH
Eingriff ohne Tumorresektion
Hyperthermie
CHI
Salvage-Radiotherapie
AUP
Brachytherapie (ungültig ab ...
Tabelle
Diagramm
Multimodal
Therapie-Phase
Therapie-Ziel
Organisations-Einheit
Therapie-Kombi
Therapie in Studie
Komplikationen
Grad der Komplikationen
Therapie-Abbruch
Therapie-Klassifikationen
Stadien d. Klassifikationen
Therapieziel
Anzahl Therapien
Kurativ
Palliativ
Diagnostisch
Adjuvant
Therapie-Ziel
Revision/Komplikation
Sonstiges
Unbekannt
Neoadjuvant
Diagnostisch
Adjuvant
Palliativ
Kurativ
Postoperativ mortality
Anastamose-insufficiency
Postoperative wound infection
Passageer AP
Emergency operation
Revision operation
Suche nach OPS oder OPS-Langname
Multiauswahl
OP-Codes (4st.)
Multiauswahl
OP-Codes (voll)
Frequency table of operations according to 4-digit OPS code
Count (OPSC)
5-60 Operationen an Prostata und Vesiculae seminales
5-40 Operationen am Lymphgewebe
5-57 Operationen an der Harnblase
5-09 -
5-01 Inzision (Trepanation) und Exzision an Schädel, Gehirn und Hirnhäuten
5-54 Andere Operationen in der Bauchregion
5-87 Exzision und Resektion der Mamma
8-13 Manipulation am Harntrakt
Frequency table of operations according to fully detailed OPS code
OPSC
OPST
Count (OPSC)
Mikrochirurgische Technik
5-984 Mikrochirurgische Technik (Zusatzkode)
5-578.40 Andere plastische Rekonstruktion der Harnblase: Harnblasenhalsplastik: Offen chirurgisch
1-511.01 Stereotaktische Biopsie am Großhirn an mehr als 5 Entnahmestellen
Stereotaktische Biopsie an intrakraniellem Gewebe: Großhirn: Mehr als 5 Entnahmestellen
5-604.12 Radikale Prostatovesikulektomie: Retropubisch, gefäß- und nervenerhaltend: Mit regionaler Lymphadenektomie
Retropubisch, gefäß- und nervenerhaltend: Mit regionaler Lymphadenektomie
Abdominale radikale Prostatektomie mit regionaler Lymphadenektomie
Abdominale radikale Prostatovesikulektomie mit regionaler Lymphade...
5-604.02
Surgery result quality
Side
Local resection status
Chirurgische Ergebnisqualität
Postoperative-Mortalität
Anastamosen-Insuffizienz
Postoperative Wundinfektion
Passagerer AP
Notfall Operation
Revisions Operation
Nullwert/Unbekannt ausblenden
Chirurgische Ergebnisq...
Anzahl Therapien
Post-OP Mort
Ja
Unbekannt
Nein
Seite
S
Unbekannt
Mittellinienzone
Beidseitig
Unpaarig
Links
Rechts
R-Status (lokal)
1(is)
2
X
1
0
Seite
Anzahl
R-Status
Anzahl
Nullwert/Unbekannt ausblenden
Nullwert/Unbekannt ausblenden

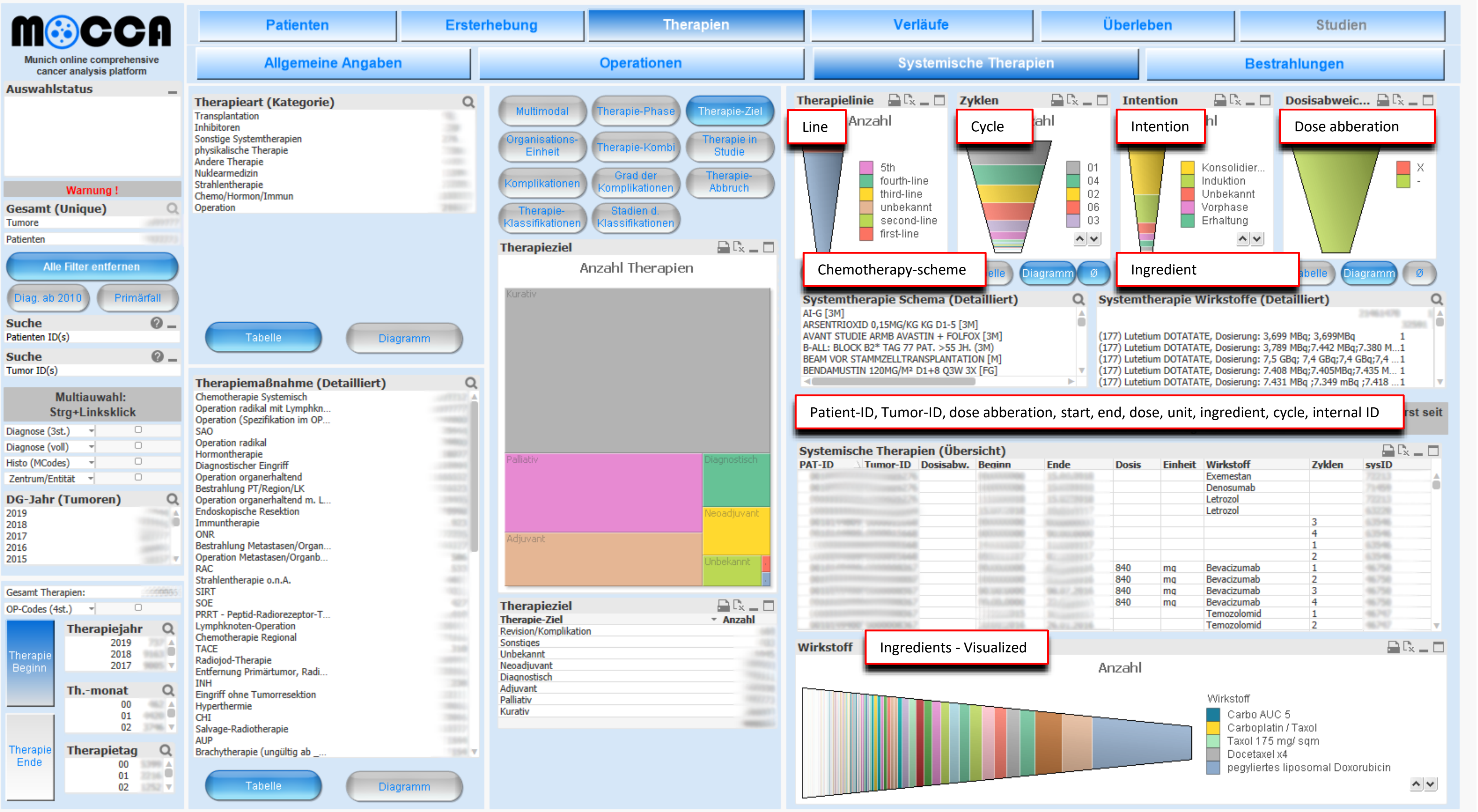

Munich online comprehensive cancer analysis platform
Patienten
Ersterhebung
Therapien
Verläufe
Überleben
Studien
Allgemeine Angaben
Operationen
Systemische Therapien
Bestrahlungen
Auswahlstatus
Warnung !
Gesamt (Unique)
Tumore
Patienten
Alle Filter entfernen
Diag. ab 2010
Primärfall
Suche
Patienten ID(s)
Suche
Tumor ID(s)
Multiauswahl: Strg+Linksklick
Diagnose (3st.)
Diagnose (voll)
Histo (MCodes)
Zentrum/Entität
DG-Jahr (Tumoren)
2019
2018
2017
2016
2015
Gesamt Therapien:
OP-Codes (4st.)
Therapiejahr
2019
2018
2017
Therapie Beginn
Th.-monat
00
01
02
Therapie Ende
Therapietag
00
01
02
Therapieart (Kategorie)
Transplantation
Inhibitoren
Sonstige Systemtherapien
physikalische Therapie
Andere Therapie
Nuklearmedizin
Strahlentherapie
Chemo/Hormon/Immun
Operation
Tabelle
Diagramm
Therapiemaßnahme (Detailliert)
Chemotherapie Systemisch
Operation radikal mit Lymphkn...
Operation (Spezifikation im OP...
SAO
Operation radikal
Hormontherapie
Diagnostischer Eingriff
Operation organerhaltend
Bestrahlung PT/Region/LK
Operation organerhaltend m. L...
Endoskopische Resektion
Immuntherapie
ONR
Bestrahlung Metastasen/Organ...
Operation Metastasen/Organb...
RAC
Strahlentherapie o.n.A.
SIRT
SOE
PRRT - Peptid-Radiorezeptor-T...
Lymphknoten-Operation
Chemotherapie Regional
TACE
Radiojod-Therapie
Entfernung Primärtumor, Radi...
INH
Eingriff ohne Tumorresektion
Hyperthermie
CHI
Salvage-Radiotherapie
AUP
Brachytherapie (ungültig ab ...
Tabelle
Diagramm
Multimodal
Therapie-Phase
Therapie-Ziel
Organisations-Einheit
Therapie-Kombi
Therapie in Studie
Komplikationen
Grad der Komplikationen
Therapie-Abbruch
Therapie-Klassifikationen
Stadien d. Klassifikationen
Therapieziel
Anzahl Therapien
Kurativ
Palliativ
Diagnostisch
Neoadjuvant
Adjuvant
Unbekannt
Therapieziel
Therapie-Ziel
Anzahl
Revision/Komplikation
Sonstiges
Unbekannt
Neoadjuvant
Diagnostisch
Adjuvant
Palliativ
Kurativ
Therapielinie
Line
Anzahl
5th
fourth-line
third-line
unbekannt
second-line
first-line
Zyklen
Cycle
01
04
02
06
03
Intention
Intention
Konsolidier...
Induktion
Unbekannt
Vorphase
Erhaltung
Dosisabweic...
Dose abberation
X
-
Chemotherapy-scheme
Diagramm
Ø
Ingredient
Diagramm
Ø
Systemtherapie Schema (Detailliert)
AI-G [3M]
ARSENTRIOXID 0,15MG/KG KG D1-5 [3M]
AVANT STUDIE ARMB AVASTIN + FOLFOX [3M]
B-ALL: BLOCK B2* TAG 77 PAT. >55 JH. (3M)
BEAM VOR STAMMZELLTRANSPLANTATION [M]
BENDAMUSTIN 120MG/M² D1+8 Q3W 3X [FG]
Systemtherapie Wirkstoffe (Detailliert)
(177) Lutetium DOTATATE, Dosierung: 3,699 MBq; 3,699MBq 1
(177) Lutetium DOTATATE, Dosierung: 3,789 MBq;7.442 MBq;7.380 M... 1
(177) Lutetium DOTATATE, Dosierung: 7,5 GBq; 7,4 GBq;7,4 GBq;7,4 ... 1
(177) Lutetium DOTATATE, Dosierung: 7.408 MBq;7.405MBq;7.435 M... 1
(177) Lutetium DOTATATE, Dosierung: 7.431 MBq ;7.349 mBq ;7.418 ... 1
Patient-ID, Tumor-ID, dose abberation, start, end, dose, unit, ingredient, cycle, internal ID
Systemische Therapien (Übersicht)
PAT-ID
Tumor-ID
Dosisabw.
Beginn
Ende
Dosis
Einheit
Wirkstoff
Zyklen
sysID
Exemestan
Denosumab
Letrozol
Letrozol
3
4
1
2
840 mg Bevacizumab 1
840 mg Bevacizumab 2
840 mg Bevacizumab 3
840 mg Bevacizumab 4
Temozolomid 1
Temozolomid 2
Wirkstoff
Ingredients - Visualized
Anzahl
Wirkstoff
Carbo AUC 5
Carboplatin / Taxol
Taxol 175 mg/ sqm
Docetaxel x4
pegyliertes liposomal Doxorubicin

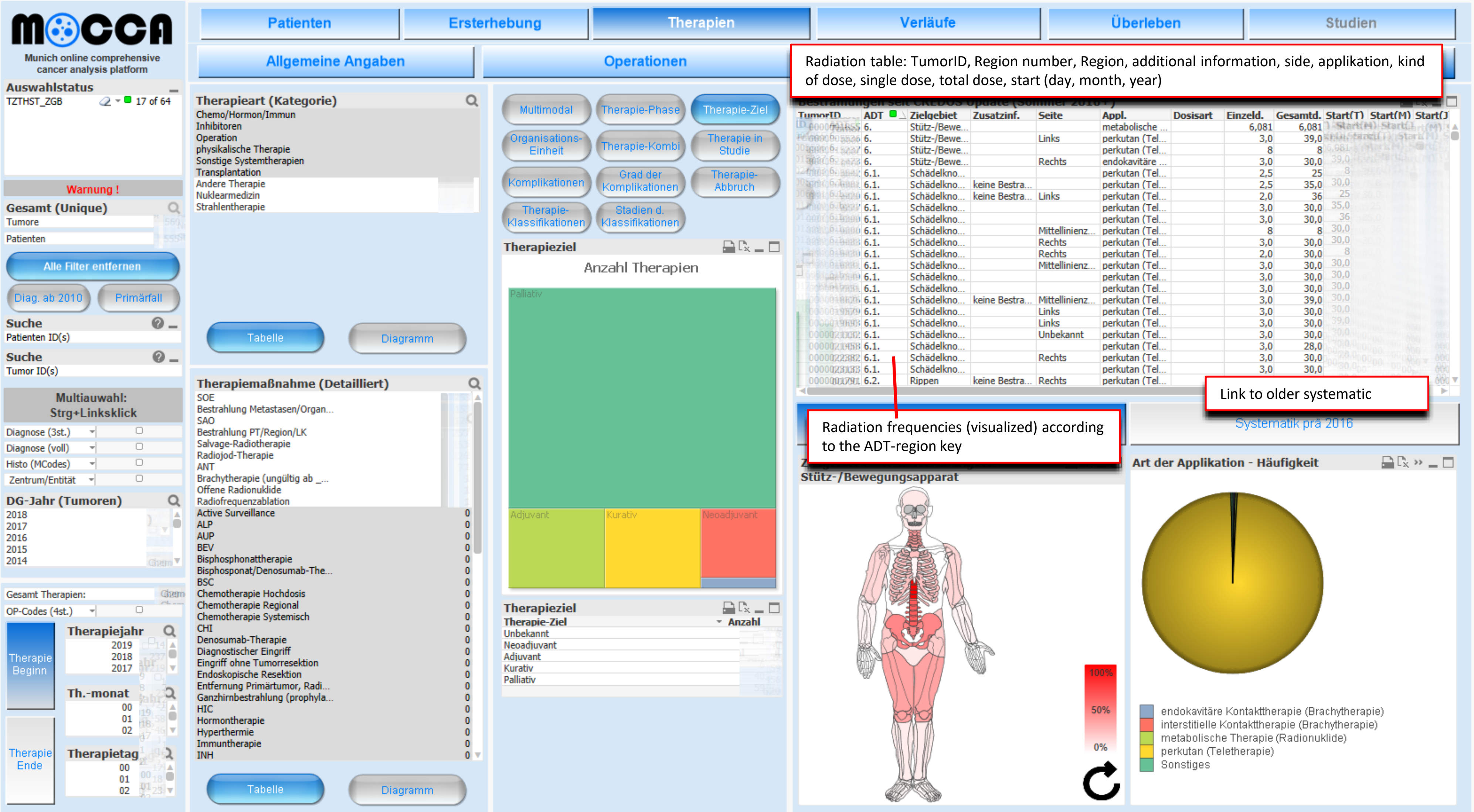
Munich online comprehensive cancer analysis platform
Patienten
Ersterhebung
Therapien
Verläufe
Überleben
Studien
Allgemeine Angaben
Operationen
Radiation table: TumorID, Region number, Region, additional information, side, applikation, kind of dose, single dose, total dose, start (day, month, year)
Link to older systematic
Systematik prä 2016
Radiation frequencies (visualized) according to the ADT-region key
Art der Applikation - Häufigkeit
Stütz-/Bewegungsapparat
Therapieziel
Anzahl Therapien
Palliativ
Adjuvant
Kurativ
Neoadjuvant
endokavitäre Kontakttherapie (Brachytherapie)
interstitielle Kontakttherapie (Brachytherapie)
metabolische Therapie (Radionuklide)
perkutan (Teletherapie)
Sonstiges
